# Molecular Mechanism of Perfluorooctane Sulfonate-Induced Lung Injury Mediated by the Ras/Rap Signaling Pathway in Mice

**DOI:** 10.3390/toxics13040320

**Published:** 2025-04-20

**Authors:** Jianhao Peng, Jinfei He, Chenglong Ma, Jiangdong Xue

**Affiliations:** College of Animal Science and Technology, Inner Mongolia University for Nationalities, Tongliao 028000, China; 18844182642@163.com (J.P.); a_286639661@163.com (J.H.); mchenglong2021@163.com (C.M.)

**Keywords:** perfluorooctane sulfonate, Ras, Rap, vascular endothelial growth factor, mitogen-activated protein kinase, signaling pathway

## Abstract

Perfluorooctane sulfonate (PFOS), a persistent organic pollutant, has raised significant public health concerns because of its widespread environmental presence and potential toxicity. Epidemiological studies have linked PFOS exposure to respiratory diseases, but the underlying molecular mechanisms remain poorly understood. Male C57 BL/6J mice were divided into a control group receiving Milli-Q water, a low-dose PFOS group (0.2 mg/kg/day), and a high-dose PFOS group (1 mg/kg/day) administered via intranasal instillation for 28 days. Lung tissue transcriptome sequencing revealed significantly enriched differentially expressed genes in the Ras and Rap signaling pathways. Key genes including Rap1b, Kras, and BRaf as well as downstream genes, such as MAPK1 and MAP2K1, exhibited dose-dependent upregulation in the high-dose PFOS exposure group. Concurrently, the downstream effector proteins MEK, ERK, ICAM-1, and VEGFa were significantly elevated in bronchoalveolar lavage fluid (BALF). These alterations are mechanistically associated with increased oxidative stress, inflammatory cytokine release, and pulmonary tissue damage. The results indicated that PFOS-induced lung injury is likely predominantly mediated through the activation of the Rap1b- and Kras-dependent BRaf-MEK-ERK axis. These findings highlight the critical role of Ras/Rap signaling pathways in PFOS-associated respiratory toxicity and underscore the need to develop therapeutic interventions targeting these pathways to mitigate associated health risks.

## 1. Introduction

Perfluorooctane sulfonate (PFOS), a persistent organic pollutant, has garnered significant attention due to its widespread environmental impact and potential health risks [1,2]. As a synthetic fluorinated compound, PFOS has been extensively used in industrial and consumer products, including firefighting foams, stain repellents, and nonstick cookware [3]. Its chemical stability and bioaccumulative properties have led to its persistence in various environmental media and biological tissues, raising concerns regarding its impact on human health [4,5]. Notably, PFOS has been identified in airborne environments, particularly in indoor settings such as air conditioner filter dust, where it can be inhaled and directly affect respiratory tissues [6,7]. This suggests that airborne indoor PFOS can enter the human body via inhalation, thereby affecting the respiratory system. Furthermore, PFOS interferes with the function of pulmonary surfactants, potentially leading to acute pulmonary toxicity and increased risk of respiratory infections [8]. Additionally, PFOS may exacerbate inflammatory responses by affecting pulmonary immune mechanisms, thereby further compromising respiratory health. According to the Global Burden of Disease, exposure to environmental pollutants such as PFOS contributes to millions of deaths annually and has a significant impact on respiratory and cardiovascular health [9,10,11]. In China, the rapid industrialization and widespread use of PFOS-containing products have resulted in elevated exposure levels, making it a critical public health issue [12,13]. Exposure to PFOS has been linked to a range of adverse health effects, particularly in the respiratory system. Owing to its small molecular size and lipophilic nature, PFOS can penetrate biological barriers, which facilitates its systemic distribution and accumulation in organs such as the lungs [14,15]. Epidemiological and experimental studies have demonstrated that both acute and chronic PFOS exposure can induce respiratory ailments, such as asthma, bronchitis, and pulmonary fibrosis, as well as exacerbate existing lung conditions [16,17,18]. PFOS disrupts the oxidative balance in the lung tissues, generating reactive oxygen species and triggering oxidative stress [19]. The process of oxidative stress sets off a sequence of inflammatory reactions, marked by the emission of pro-inflammatory cytokines such as tumor necrosis factor-alpha (TNF-α), interleukin-6 (IL-6), and vascular endothelial growth factor (VEGF). These biochemical substances play a role in promoting chronic inflammatory states and contributing to tissue harm [20,21].

Molecular mechanisms underlying PFOS-induced lung injury are complex. Previous studies have identified several signaling pathways involved in PFOS toxicity, including oxidative stress, inflammation, and apoptosis [22,23]. Notably, the Ras and Rap signaling pathways have emerged as critical regulators of cellular responses to environmental stressors such as PFOS exposure [24,25]. These pathways play essential roles in cell proliferation, differentiation, and survival, and their dysregulation has been implicated in the pathogenesis of lung diseases, including fibrosis and chronic obstructive pulmonary disease (COPD) [26,27]. For instance, PFOS exposure activates the JAK-STAT signaling pathway, which exacerbates Th2-mediated airway inflammation, a hallmark of asthma [28]. Additionally, maternal PFOS exposure can alter the epigenetic landscape in fetal tissues, potentially predisposing the offspring to metabolic and inflammatory disorders [29].

In this study, we focused on the role of the Ras and Rap signaling pathways in PFOS-induced lung injury. Using transcriptome sequencing data from the lung tissues of PFOS-exposed and control mice, we investigated the expression of genes associated with these pathways. We further examined the expression of related genes in lung tissues and protein levels in the bronchoalveolar lavage fluid (BALF). Our findings suggest that PFOS exposure disrupts the oxidative balance, amplifies inflammatory responses, and dysregulates Ras/Rap signaling, collectively contributing to pulmonary damage. These findings highlight the need for targeted interventions to mitigate PFOS-associated respiratory risks.

## 2. Material and Method

### 2.1. Ethics

Animal experimentation was carried out in strict compliance with global ethical norms governing animal-based research. Prior to the commencement of the study, the research plan had received authorization from the Medical Ethics Committee of the Affiliated Hospital of Inner Mongolia University for Nationalities. The approval code for this protocol was NM-LL-2024-06-14-01.

### 2.2. PFOS Sample Preparation and Characterization

Perfluorooctane sulfonate (PFOS, potassium salt; purity ≥ 98%, purchased from Sigma-Aldrich, St. Louis, MO, USA) was dissolved in Milli-Q water (with a resistivity of 18.2 MΩ·cm) to prepare a primary stock solution with a nominal concentration of 0.5 g/L. Using Milli-Q water as the solvent, the stock solution was serially diluted to obtain working solutions with target concentrations of 0.2 and 1 mg/kg/day to improve solubility and ensure uniform dispersion. To confirm the precision of PFOS concentrations, an analytical approach was employed. All solutions underwent examination via ultra-high performance liquid chromatography tandem mass spectrometry (UHPLC—MS/MS). The equipment utilized consisted of a Vanquish Flex liquid chromatograph and a TSQ Altis mass spectrometer, both products of Thermo Fisher Scientific (Waltham, MA, USA).

### 2.3. Animal and Exposure Protocol

Male C57 BL/6J mice at seven weeks of age were procured from the Laboratory Animal Center affiliated with the Chinese Academy of Medical Sciences in Beijing, China. These animals were provided with a standard laboratory chow. The environmental conditions for housing the mice were set at approximately 24 °C, with a regular 12-h light and 12-h dark cycle, along with controlled humidity levels. Additionally, the mice had unrestricted access to drinking ultrapure water. Before the experiment, the mice were acclimated for one week. The mice were randomly divided into three groups of five mice (N = 15) according to the following conditions: (1) administered Milli-Q water (vehicle control) daily via intranasal instillation; (2) administered PFOS solution at 0.2 mg/kg body weight/day via intranasal instillation; and (3) PFOS solution was administered at 1 mg/kg body weight/day via intranasal instillation. Mice that received intranasal Milli-Q water were used as the control group. The mice receiving intranasal instillation of the working solution at a dose of 0.2 mg/kg/day were defined as the low-dose PFOS exposure group, and those receiving the working solution at a dose of 1 mg/kg/day were defined as the high-dose PFOS exposure group. Mice in each experimental group were subjected to daily intranasal instillations of the corresponding treatments for a continuous period of 28 days. This exposure protocol was meticulously designed with reference to previously published studies that investigated the toxicological effects of PFOS [30]. The doses selected (0.2 and 1 mg/kg/day) and exposure duration (28 days) were chosen to mirror the sub-acute toxicity thresholds [31], in line with well-established models employed to evaluate the health effects induced by PFOS, such as hepatotoxicity, immunotoxicity, and metabolic disruption [32]. All groups were provided with a normal diet and clean water. After the PFOS exposure period, all experimental animals were euthanized via intraperitoneal injection of sodium pentobarbital (0.1 mg/g). Lung tissues were rinsed with pre-cooled PBS (0.1 M, pH 7.4) to remove blood residues. The collected BALF and lung tissues were quickly frozen with liquid nitrogen and then stored in an ultra-low temperature refrigerator at −80 °C for subsequent analysis.

### 2.4. RNA-Seq

Total RNA extraction from frozen lung tissues was performed using a DNA/RNA Isolation Kit (Tiangen Biotech, Beijing, China), adhering strictly to the manufacturer’s specified protocol. The integrity of the isolated RNA was evaluated via an Agilent 2100 Bioanalyzer (Agilent Technologies., Santa Clara, CA, USA), with samples meeting an RNA Integrity Number (RIN) threshold greater than 8.0. Quantification of RNA concentration was subsequently conducted using a NanoDrop 2000 spectrophotometer (Thermo Scientific, Waltham, MA, USA) to ensure suitable material for downstream applications. Strand-specific RNA sequencing libraries were generated through the use of the NEBNext Ultra II RNA Library Prep Kit, following standardized procedures for library construction. These libraries were then subjected to high-throughput sequencing on an Illumina NovaSeq 6000 platform (Illumina, San Diego, CA, USA), producing 150 bp paired-end reads to capture transcriptomic profiles comprehensively. To identify genetic alterations across experimental groups, a comparative transcriptomic analysis was undertaken to detect differentially expressed genes among control, high-dose, and low-dose treatment groups. This analysis was followed by functional enrichment studies, incorporating Gene Ontology (GO) and Kyoto Encyclopedia of Genes and Genomes (KEGG) pathway analyses, to characterize biological processes impacted by experimental conditions. Gene expression levels were systematically quantified using transcripts per million (TPM) as a normalized metric, enabling precise mapping of transcriptional changes within pulmonary tissue samples.

### 2.5. Real-Time PCR

Total RNA was extracted from tissue samples using a DNA/RNA Isolation Kit (Tiangen Biotech, Beijing, China) according to the manufacturer’s protocol. RNA integrity was verified by agarose gel electrophoresis, and RNA concentration was quantified using a NanoDrop 2000 spectrophotometer (Thermo Scientific, Waltham, MA, USA). To perform quantitative real-time PCR (qPCR) analysis, cDNA synthesis was conducted from 1 μg of total RNA using the PrimeScript RT Reagent Kit (Takara, Tokyo, Japan). Triplicate qPCR reactions were set up with Maxima SYBR Green qPCR Master Mix (CWBIOtech, Beijing, China), utilizing an ABI Prism 7500 Sequence Detection System (Applied Biosystems, Waltham, MA, USA). Thermal cycling parameters included an initial 10 min denaturation at 95 °C, followed by 45 cycles consisting of 25 s at 95 °C, 1 min at 60 °C, and 10 s at 72 °C. β-Actin was used as the internal reference gene for normalization, and primer sequences (designed by BGI, Shenzhen, China) are listed in Table 1. A melting curve analysis was performed to confirm the specificity of the amplification products.

### 2.6. Determination of MEK and ERK Protein Expression Levels

Quantification of the concentrations of mitogen-activated protein kinase (MEK) and extracellular signal-regulated kinase (ERK) in bronchoalveolar lavage fluid (BALF) was carried out with the help of commercial ELISA kits from Gersion Biotechnology in Beijing, China. This process was carried out following the detailed protocol provided by the kit manufacturer. Before the quantification, the BALF samples were first centrifuged. The centrifugation was performed at a speed of 1000× *g* for a duration of 10 min, and the temperature was maintained at 4 °C. Duplicate runs were conducted for each assay, with absorbance measured at 450 nm using a SpectraMax M3 microplate reader (Molecular Devices, San Jose, CA, USA).

### 2.7. Determination of ICAM-1 and VEGFa Protein Expression Levels

The levels of ICAM-1 and VEGFa in bronchoalveolar lavage fluid (BALF) were evaluated using commercially available ELISA kits (Gersion Biotechnology, Beijing, China), with all procedures conducted in strict accordance with the manufacturer’s instructions. Prior to analysis, BALF samples were centrifuged at 1000× *g* for 10 min at 4 °C, after which the supernatants were carefully collected for subsequent processing. To maintain precision, each assay was performed in duplicate. Absorbance measurements at 450 nm were obtained using a SpectraMax M3 microplate reader (Molecular Devices, USA), providing reliable data for subsequent statistical analysis.

### 2.8. Measurement of Inflammatory Factors in BALF

The levels of IL-1β, TNF-α, IL-6, and IL-8 in bronchoalveolar lavage fluid (BALF) were evaluated using commercially available ELISA kits (Gersion Biotechnology, Beijing, China), with all procedures executed in strict adherence to the manufacturer’s detailed instructions. Prior to analysis, BALF samples underwent centrifugation at 1000× *g* for 10 min at 4 °C, after which supernatants were carefully collected for subsequent assay processing. To maintain analytical reliability, each detection step was performed in duplicate. Absorbance measurements at 450 nm were obtained using a SpectraMax M3 microplate reader (Molecular Devices, USA), providing consistent and reproducible data for subsequent quantitative analysis.

### 2.9. Statistical Analysis

The results are reported as means ± standard error of the mean (SEM). Data normality was verified using Shapiro–Wilk tests (α = 0.05). Group comparisons were performed using one-way ANOVA. Statistical significance was set at *p* < 0.05. All analyses were performed using GraphPad Prism 8.0.

## 3. Result

### 3.1. Effects of PFOS Exposure on Inflammatory Factors Expression

**Figure 1:**
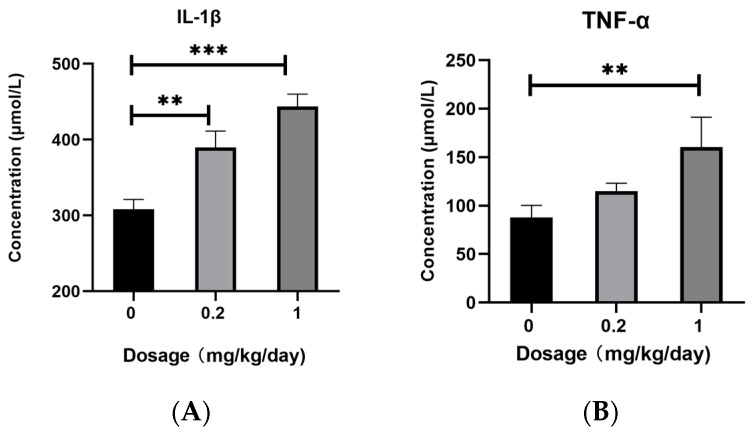

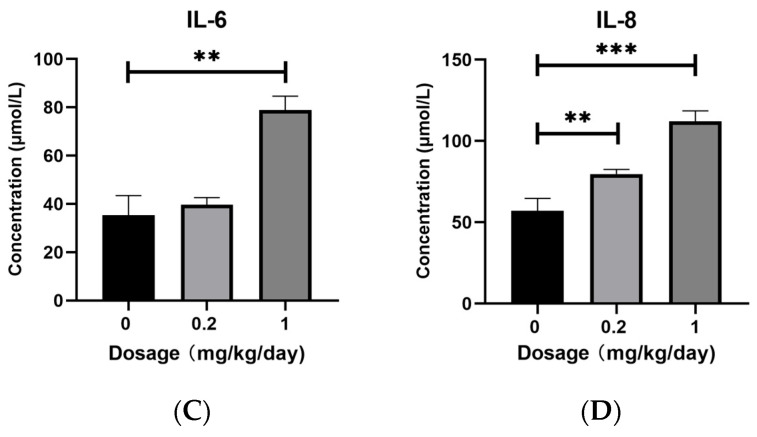
Enzyme-linked Immunosorbent Assay (ELISA). (**A**) The protein expression level of IL-1β in BALF. (**B**) The protein expression level of TNF-α in BALF. (**C**) The protein expression level of in IL-6 BALF. (**D**) The protein expression level of IL-8 in BALF. All data are presented as mean ± SEM. (N = 5, * *p* < 0.05, ** *p* < 0.01, *** *p* < 0.001).

We quantitatively analyzed the protein expression levels of IL-1β, TNF-α, IL-6, and IL-8 in mouse BALF using commercially available ELISA kits. As shown in Figure 1A,D, low-dose PFOS exposure induced a significant upregulation of IL-1β and IL-8 protein expression. High-dose PFOS exposure resulted in markedly elevated levels of all detected proteins, including IL-1β, TNF-α, IL-6, and IL-8 (Figure 1). Notably, the protein levels of IL-1β, TNF-α, IL-6, and IL-8 exhibited a dose-dependent increase, with higher PFOS concentrations causing more pronounced upregulation.

### 3.2. PFOS Exposure Leads to Changes in Signaling Pathways

**Figure 2:**
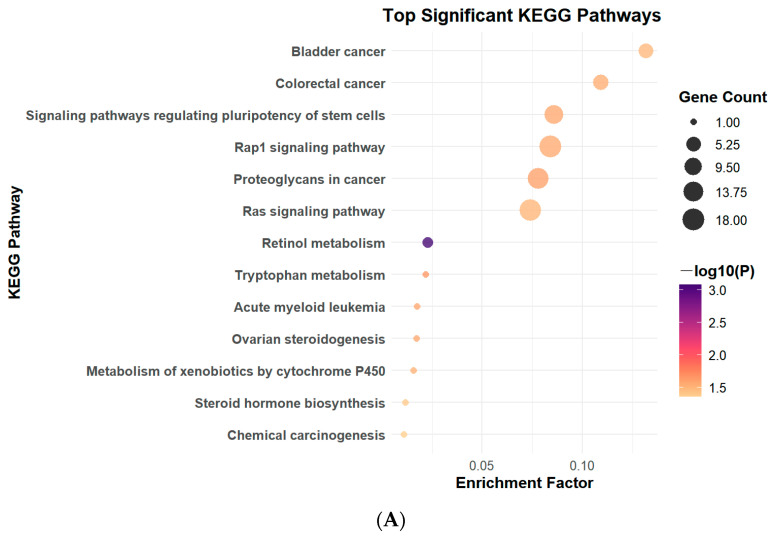

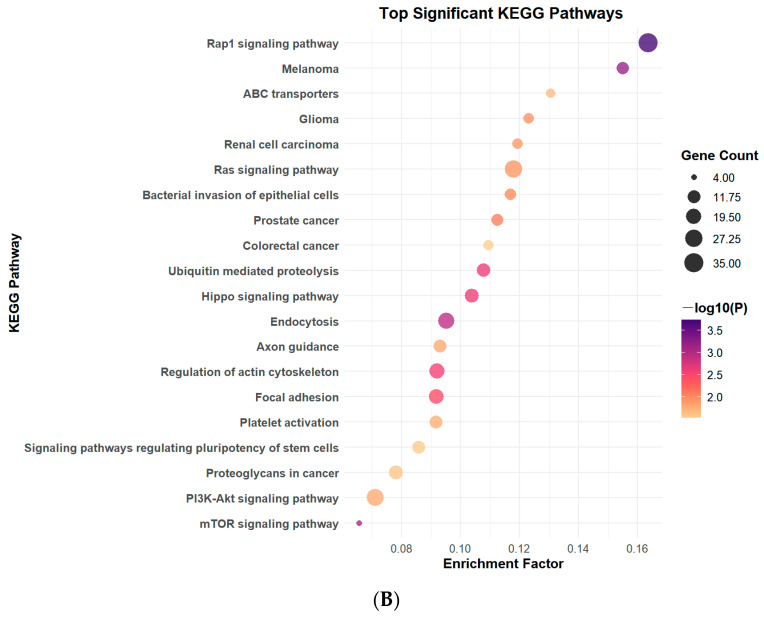
Top Significantly Enriched KEGG Pathways. (**A**) KEGG pathway enrichment in low-dose PFOS exposure group. (**B**) KEGG pathway enrichment in high-dose PFOS exposure group.

Findings from KEGG pathway analysis indicated that the Rap signaling pathway exhibited notable upregulation in the low-dose PFOS group when compared with the control group (Figure 2A). In the high-dose PFOS group, this upregulation became even more pronounced, with 28 genes within the pathway—including Rap1b, Kras, and BRaf—displaying significant expression alterations (Figure 2B). These results indicate that PFOS exposure can significantly induce alterations in the Rap signaling pathway in mice, and the associated changes in this pathway become more prominent with increasing PFOS concentrations. Furthermore, among the pathways related to Rap, the mitogen-activated protein kinase (MAPK) pathway exhibited significant changes. The results of gene ontology analysis and differential expression analysis are detailed in the Supplementary Materials.

### 3.3. Effects of PFOS Exposure on Gene Expression

**Figure 3:**
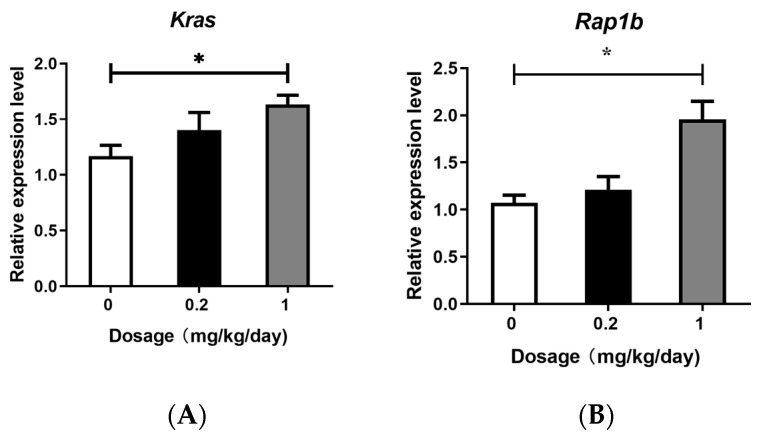

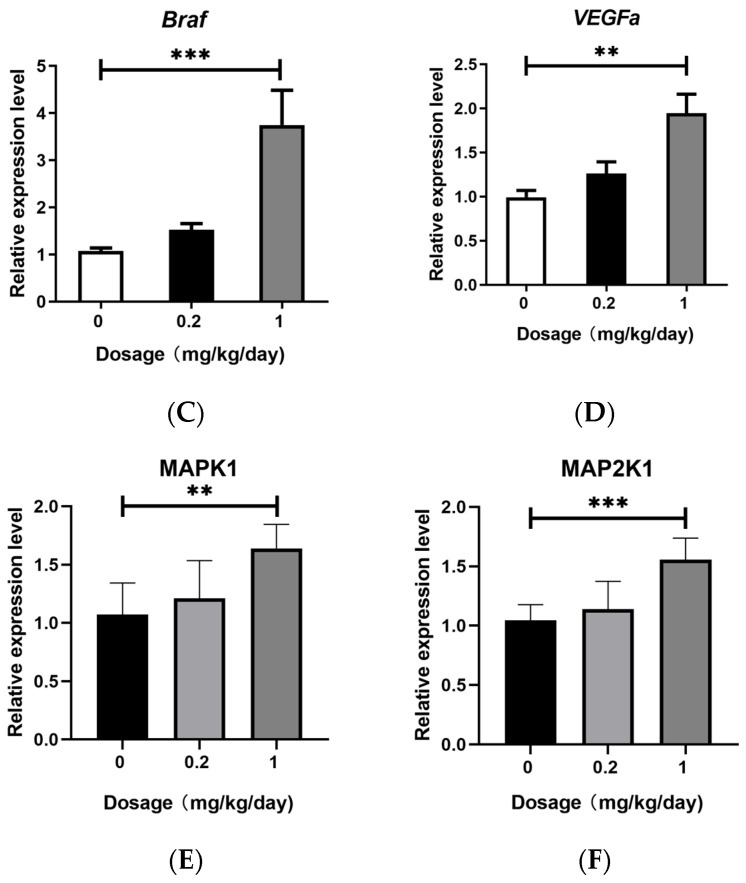
Quantitative real-time polymerase chain reaction (q-PCR). (**A**) The gene expression level of Kras in lung tissues. (**B**) The gene expression level of Rap1b in lung tissues. (**C**) The gene expression level of BRaf in lung tissues. (**D**) The gene expression level of VEGFa in lung tissues. (**E**) The gene expression level of MAPK1 in lung tissues. (**F**) The influence on the gene expression level of MAP2K1 in lung tissues. All data are presented as mean ± SEM. (N = 5, * *p* < 0.05, ** *p* < 0.01, *** *p* < 0.001).

The RAS gene family comprises three isoforms, H-RAS, K-RAS, and N-RAS, with K-RAS being the most influential in human lung cancer [31,32,33]. RAP has two isoforms: RAP1A and RAP1B [34]. The RAF protein, a critical component of the Ras/Raf/MEK/ERK signaling cascade, consists of three isoforms: C-RAF, B-RAF, and A-RAF. Among these, B-RAF is widely recognized in cancer owing to its frequent mutations in tumors and its highest basal activity compared to other isoforms [35]. To investigate the effects of PFOS exposure on these signaling molecules, we quantified the expression levels of VEGF, RAP, RAS, and RAF isoforms (VEGFa, Rap1b, Kras, and BRaf), as well as the downstream pathway-related genes MAPK1 and MAP2K1, using quantitative PCR. The results demonstrated that high-dose PFOS exposure significantly upregulated the expression of Kras, Rap1b, BRaf, VEGFa, MAPK1, and MAP2K1 (Figure 3). In contrast, no significant changes in the expression levels of these genes were observed in the low-dose PFOS exposure group, although a slight upward trend compared with the control group was noted.

### 3.4. Effects of PFOS Exposure on Downstream Signaling Pathway

**Figure 4:**
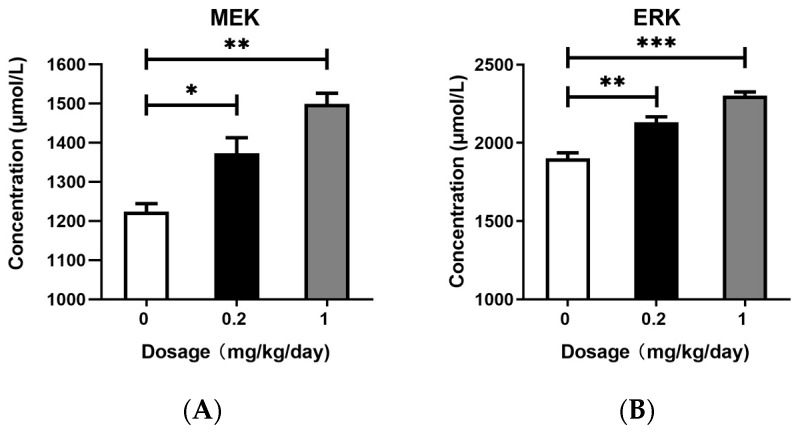
Enzyme-linked Immunosorbent Assay (ELISA). (**A**) The protein expression level of MEK in BALF. (**B**) The protein expression level of ERK in BALF. All data are presented as mean ± SEM. (N = 5, * *p* < 0.05, ** *p* < 0.01, *** *p* < 0.001).

We quantitatively analyzed the protein expression levels of MEK and ERK in mouse BALF using commercially available ELISA kits. As shown in Figure 4A,B, PFOS exposure induced the significant upregulation of ERK and MEK protein expression compared with that in the control group. Notably, ERK and MEK increased in a dose-dependent manner, with higher PFOS concentrations resulting in more pronounced upregulation.

### 3.5. Effects of PFOS Exposure on ICAM-1 and VEGFa Expression

**Figure 5:**
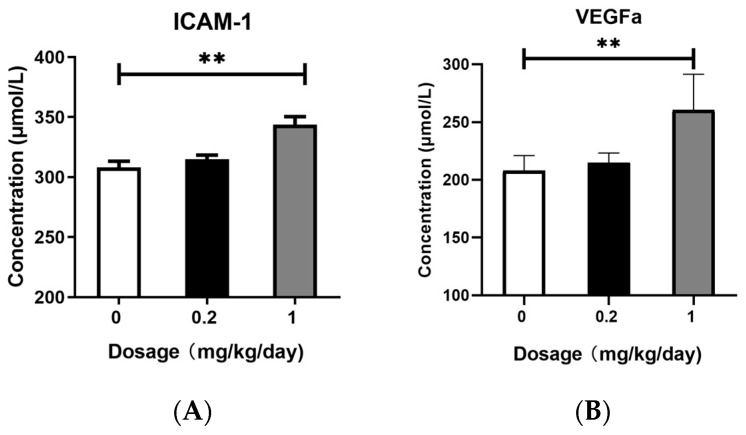
Enzyme-linked Immunosorbent Assay (ELISA). (**A**) The protein expression level of ICAM-1 in BALF. (**B**) The protein expression level of VEGFa in BALF. All data are presented as mean ± SEM. (N = 5, * *p* < 0.05, ** *p* < 0.01, *** *p* < 0.001).

We quantitatively analyzed the protein expression levels of ICAM-1 and VEGFa in mouse BALF using commercially available ELISA kits. As shown in Figure 5, after low-dose PFOS exposure, there were no obvious changes in the protein levels of ICAM-1 and VEGFa in BALF. However, after high-dose PFOS exposure, the protein levels of ICAM-1 and VEGFa in BALF increased significantly.

## 4. Discussion

In this study, we demonstrated that PFOS exposure induced significant dysregulation of the Ras and Rap signaling pathways in murine lung tissues, accompanied by amplified oxidative stress, inflammatory cytokine release, and pulmonary damage. Transcriptomic analysis revealed that the Rap signaling pathway was markedly upregulated in both the low- and high-dose PFOS-exposed groups, with high-dose exposure eliciting more pronounced effects. Key genes in this pathway, including Kras, Rap1b, and BRaf, as well as the downstream genes MAPK, MAP2K1, and VEGFa, were overexpressed in a dose-dependent manner. Furthermore, downstream effector proteins such as ERK, MEK, and inflammatory factors IL-1β, TNF-α, IL-6, and IL-8 were significantly elevated in BALF. Moreover, the increase in the expression levels of ICAM-1 and VEGFa proteins in the BALF further reveals the possibility of tissue damage. These findings suggest that PFOS disrupts cellular signaling cascades, exacerbating lung injury through the interplay between oxidative stress and inflammation, as well as aberrant activation of rap-associated pathways.

In this study, our KEGG enrichment analysis revealed that both the Ras and Rap signaling pathways were significantly upregulated in the PFOS-exposed groups, with higher doses of PFOS exposure eliciting more pronounced effects. Ras and Rap GTPases act as molecular switches that govern cellular responses to environmental stressors, including oxidative and inflammatory stimuli. Our findings align with those of previous studies linking PFOS exposure to oxidative imbalance and inflammatory cytokine release in lung tissues [36,37]. Moreover, our qPCR results demonstrated that the gene expression levels of Rap1b, Kras, and BRaf in PFOS-exposed group mice were significantly upregulated compared to the control group. These findings validate the transcriptomic sequencing analyses and further suggest that PFOS stimulation promotes the substantial binding of Rap1b and RAS to GTP [38,39], thereby activating downstream effector molecules such as BRaf [40]. This mechanism may drive abnormal cellular proliferation and differentiation. Although Ras and Rap are highly homologous proteins that function in distinct yet interconnected signaling networks, they can both bind to RAF and regulate RAS downstream signaling pathways [41]. Previous studies have demonstrated that RAP can directly activate BRaf independently of RAS, bypassing the classical Ras–Raf interaction [39]. Our findings indicate that under PFOS exposure, both Rap1b and Kras act on BRaf and play a predominant role in downstream signal transduction.

Activated BRaf stimulates downstream effector kinases and participates in the regulation of the downstream MEK pathway, thereby modulating intercellular proliferation, differentiation, and inflammatory responses [36,37,42]. The MAPK signaling pathway plays a pivotal role in regulating cell growth, development, division, and death, with the ERK/MAPK pathway playing a critical role in these processes. ERK, a key member of the MAPK family, is activated by its association with RAF/MEK, and phosphorylated MEK further activates ERK. Once activated, ERK modulates transcription factors and enzymes involved in cell proliferation, differentiation, transformation, and apoptosis, ultimately contributing to the development of inflammation, cancer, and other diseases [35,43,44]. The outcomes of our qPCR indicated that, in comparison with the control group, the high-dose PFOS exposure group showed a significant increase in the expression levels of MAPK1, MAP2K1, and VEGFa. To confirm these results, we employed ELISA to measure the concentrations of ERK and MEK in BALF. The data obtained from the ELISA showed that mice in the high-dose PFOS exposure group had notably higher levels of MEK and ERK than those in the control group. The heightened expression of genes and proteins associated with the MAPK pathway suggests that the downstream signaling cascade of this pathway remained in an activated state. In this study, we also examined the protein expression levels of key inflammatory cytokines IL-1β, IL-6, TNF-α, and IL-8, as well as the endothelial homeostasis-related factors ICAM-1 and VEGFa in BALF. The results demonstrated that the levels of IL-1β, IL-6, TNF-α, IL-8, ICAM-1, and VEGFa were significantly elevated in the high-dose PFOS-exposed group compared to the control group. Activated ERK further promotes the transcriptional activation of inflammatory mediators such as ICAM-1 and VEGFa, exacerbating vascular permeability and leukocyte adhesion [33,38,45,46,47], which is consistent with the observed PFOS-induced oxidative stress and cytokine release. The dose-dependent increase in VEGFa and ICAM-1 expression further underscores PFOS’s role in disrupting endothelial homeostasis, thereby promoting chronic inflammation and tissue remodeling. Notably, some studies have reported that Rap plays a critical role in cell adhesion, with B cell adhesion regulated by Rap GTPases [48]. These findings suggest that PFOS exposure may disrupt the Ras/Rap signaling pathway, leading to the activation of redox-sensitive kinases such as MAPK. This activation amplified the inflammatory cascade, resulting in sustained lung injury.

Although our study elucidated the impact of PFOS on Ras/Rap signaling, several limitations warrant consideration. Murine models may not fully reflect human exposure dynamics. Additionally, the short-term (28-day) exposure period studied here limits insights into long-term carcinogenic risks, despite evidence linking Ras/Rap dysregulation to pulmonary fibrosis and malignancy [35,43]. Future studies should explore epigenetic modifications and crosstalk with other pathways, such as JAK-STAT, to comprehensively map PFOS toxicity and examine exposure over longer periods.

## 5. Conclusions

This study delineates the pivotal role of the Ras/Rap signaling pathway in mediating PFOS-induced pulmonary injury, driven by the activation of the Rap1b/Kras-BRaf-MEK-ERK axis. Chronic PFOS exposure triggered dose-dependent dysregulation of key signaling molecules, including Rap1b, Kras, and BRaf, alongside downstream effectors (MAPK1, MAP2K1, and VEGFa), which collectively exacerbated oxidative stress and inflammatory cascades. Elevated levels of pro-inflammatory cytokines (IL-1β, TNF-α, IL-6, and IL-8) and adhesion molecules (ICAM-1 and VEGFa) in bronchoalveolar lavage fluid underscored the interplay between sustained ERK/MAPK activation and pulmonary tissue damage. Notably, the dose–response relationship highlighted the environmental relevance of low-dose chronic exposure, emphasizing its potential public health risks. These findings advance mechanistic insights into PFOS toxicity and propose Ras/Rap signaling as a therapeutic target for mitigating respiratory damage. Future studies should explore long-term carcinogenic risks, epigenetic modifications, and translational interventions, such as MEK/ERK inhibitors or antioxidants, to bridge experimental models with human pathophysiology. This work underscores the urgency of stricter PFOS regulation and biomonitoring in high-risk populations, offering a foundation for combating PFOS-associated respiratory diseases.

## Figures and Tables

**Table 1:** Sequence of primers used for Real-time PCR.

Gene	Sequence of Primer (5′→3′)	
	Forward	Reverse
	C57 BL/6J Mice	
***R*** ***ap*** ***-1*** ***b***	TGCAGGAACGGAGCAATTCA	GTCGACTGTGCTGTGATGGA
***B*** ***r*** ***af***	AGTGGTACCCGCAAGATGTG	TGGTTTCTTCTCTCCATCCTGA
***V*** ***egf*** ***a***	CGGGCCTCGGTTCCA	GCAGCCTGGGACCACTTG
***Kras***	GACACGAAACAGGCTCAGGA	GCATCGTCAACACCCTGTC
***M*** ***apk*** ***1***	CCCAAGTGATGAGCCCATTG	CTTACACCATCTCTCCCTTGCT
***M*** ***ap*** ***2*** ***k*** ***1***	CACCAGAGGGAAGCTTGAGAT	GCCTCCAGGTTGGTCCTCTC
***β-Actin***	CACTGTCGAGTCGCGTCC	TCATCCATGGCGAACTGGTG

## Data Availability

The data supporting the conclusions of this article are available from the corresponding author upon reasonable request. This study did not involve human research.

## Supplementary Materials

The following supporting information can be downloaded at: https://www.mdpi.com/article/10.3390/toxics13040320/s1, Figure S1:The GO analysis; Figure S2: Analysis of gene expression differences.
