# Supplementary material for: Molecular Mechanism of Perfluorooctane Sulfonate-Induced Lung Injury Mediated by the Ras/Rap Signaling Pathway in Mice
Source: Toxics. 2025 Apr 20;13(4):320. doi: 10.3390/toxics13040320 (PMC12030834; doi:10.3390/toxics13040320)
Supplement: Supplementary file 1 [file toxics-13-00320-s001.zip › toxics-3561643-supplementary.pdf]

Supplementary Material

RNA extraction methodology

(1) Place 50 mg of mouse lung tissue into a 1.5 mL centrifuge tube, add an appropriate amount of liquid nitrogen, and fully homogenize the tissue using a tissue homogenizer. Then, add 0.5 mL of Trizol solution and place the tube on ice for half an hour. Next, centrifuge the tube at 12,000 r/min for 10 min using a centrifuge. Discard the precipitate and transfer the supernatant to a new 1.5 mL centrifuge tube.
(2) Add 200 μL of chloroform to the centrifuge tube. After thorough mixing, store the tube on ice and wait for 15 min. Then, centrifuge it at 12,000 r/min for 20 min. Observe that the sample separates into three layers, and RNA is mainly present in the upper layer. Then, transfer the upper colorless aqueous phase to a new 1.5 mL centrifuge tube.
(3) Add 500 μL of pre - cooled isopropanol to the upper colorless aqueous phase. Gently mix the solution and place it on ice for 30 min or longer to allow the RNA to fully precipitate.
(4) Centrifuge the tube at 12,000 r/min for 15 min at 4°C using a high - speed refrigerated centrifuge. After centrifugation, a transparent RNA precipitate can be seen at the bottom of the tube. Carefully pour off the supernatant and keep the precipitate at the bottom of the centrifuge tube.
(5) Add 500 μL of a 75% ethanol solution prepared with DEPC - treated water to the precipitate. Use a pipette to slowly pipette the solution up and down to resuspend the precipitate. Then, centrifuge the tube at 12,000 r/min for 5 min at 4°C using a high - speed refrigerated centrifuge. Discard the supernatant and repeat the above steps once.
(6) After allowing the ethanol to evaporate completely at room temperature, dissolve the RNA with 50 μL of DEPC - treated water.

(7) Measure the RNA concentration of the sample and the OD260/280 ratio using a Nanodrop to evaluate the quality of the RNA.

(8) Freeze the RNA samples in a -80°C refrigerator for subsequent analysis.

Supplementary Figure

The GO analysis method was used to study the roles of genes affected by perfluorooctane sulfonate (PFOS) in cellular components (CC), molecular functions (MF), and biological processes (BP). The results of the top 10 significantly enriched CC, MF, and BP after acute PFOS exposure are shown in Supplementary Figures 1A, 1B, and 1C. Briefly, several processes related to protein catabolic processes and regulatory activities of nucleoside triphosphatases were altered after PFOS exposure.

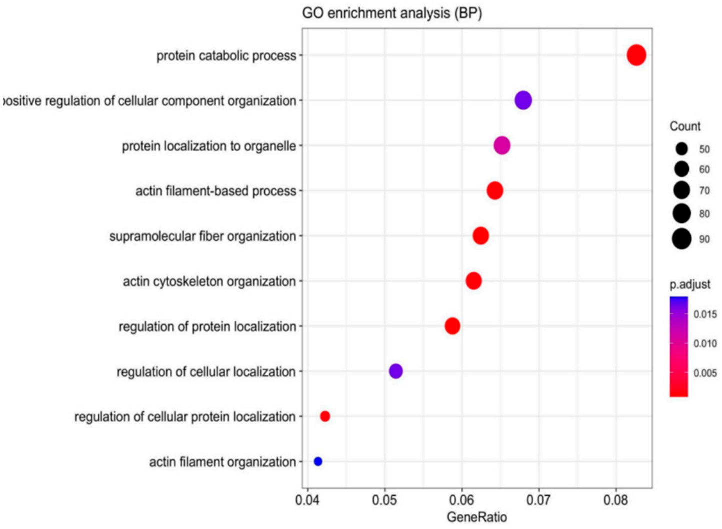

(A)

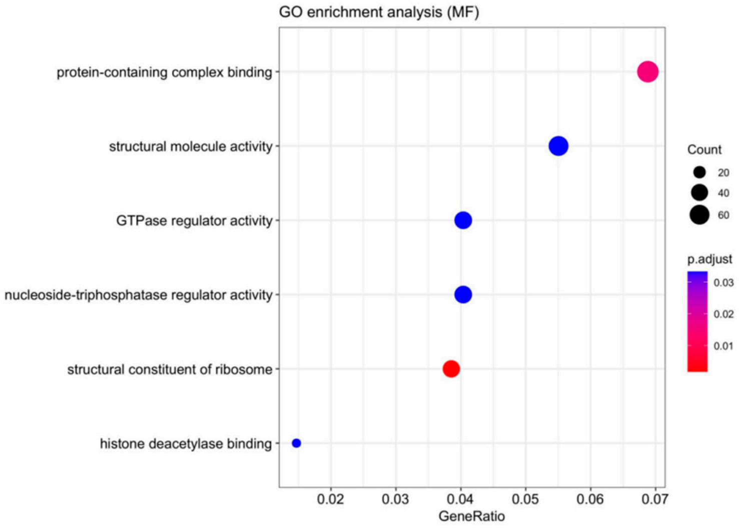

(B)

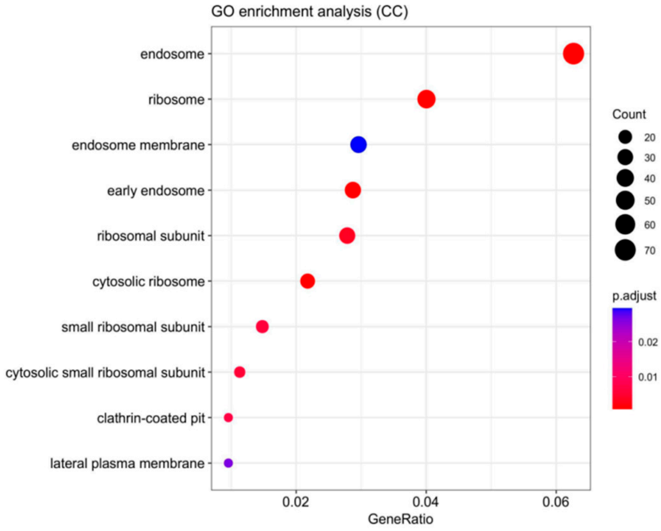

(C)

Supplementary Figure S1. Lung transcription sequencing results. (A) Top 10 GO enrichment analysis (BP). (B) Top 10 GO enrichment analysis (MF). (C) Top 10 GO enrichment analysis (CC)

The volcano plot was used to investigate changes in gene expression levels affected by PFOS. As shown in Supplementary Figure 2A, transcriptome sequencing of mouse lungs revealed that after low-dose PFOS exposure, the expression of Rap1b, Kras, and Braf genes was significantly upregulated. Supplementary Figure 2B further indicated that after high-dose PFOS exposure, the changes in the expression levels of Rap1b, Kras, and Braf genes became more pronounced.

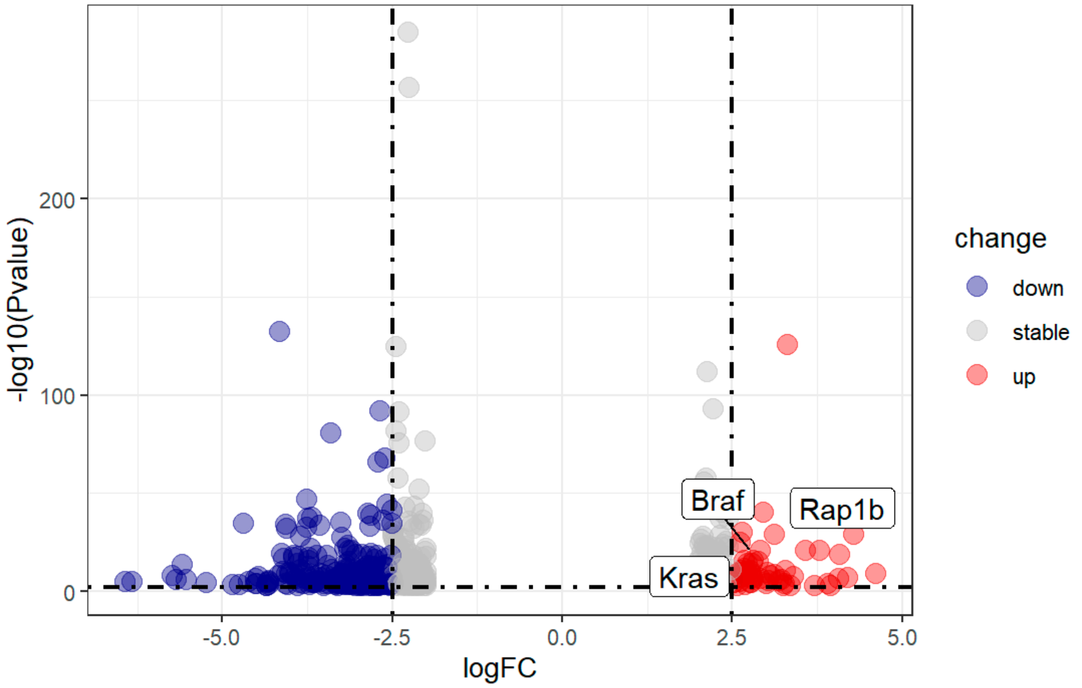

(A)

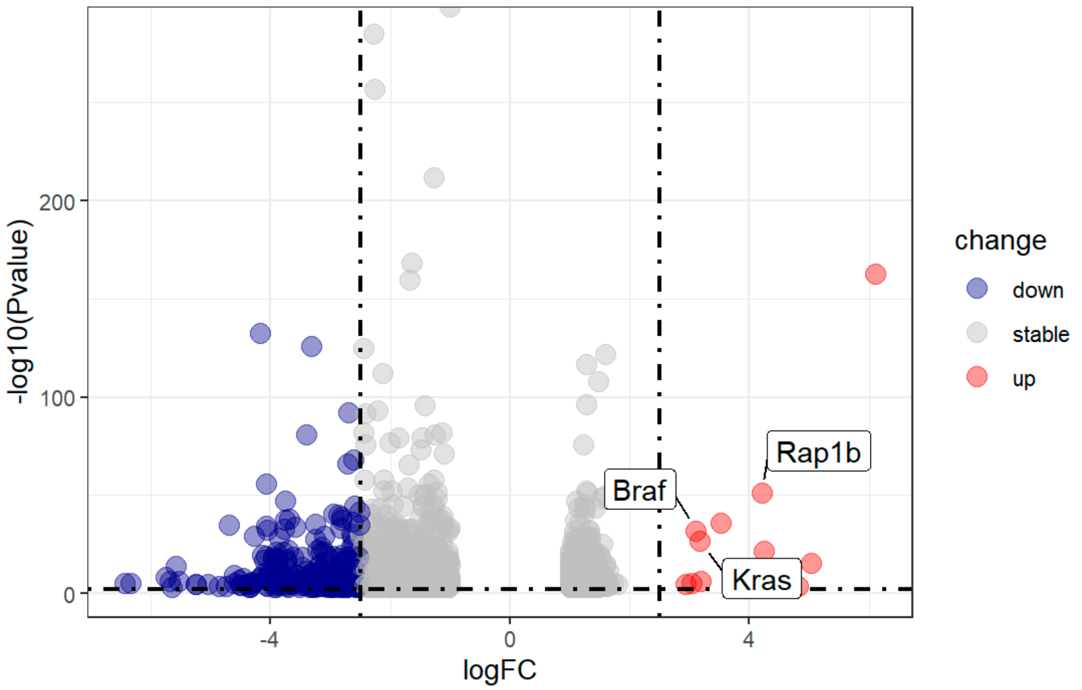

(B)

Supplementary Figure S2. Lung transcription sequencing results. (A) Analysis of differentially expressed genes in the low-dose PFOS exposure group. (B) Analysis of differentially expressed genes in the high-dose PFOS exposure group.
